# Roxadustat, a Hypoxia-Inducible Factor 1α Activator, Attenuates Both Long- and Short-Term Alcohol-Induced Alcoholic Liver Disease

**DOI:** 10.3389/fphar.2022.895710

**Published:** 2022-05-10

**Authors:** Yongyao Gao, Xiaomeng Jiang, Daigang Yang, Wentong Guo, Dandan Wang, Ke Gong, Ying Peng, Hong Jiang, Cunyuan Shi, Yajun Duan, Yuanli Chen, Jihong Han, Xiaoxiao Yang

**Affiliations:** ^1^ Key Laboratory of Metabolism and Regulation for Major Diseases of Anhui Higher Education Institutes, College of Food and Biological Engineering, Hefei University of Technology, Hefei, China; ^2^ Zhejiang Jianfeng Pharmaceutical Co., Ltd., Jinhua, China; ^3^ School of Pharmacy, Anhui University of Chinese Medicine, Hefei, China; ^4^ College of Life Sciences, Key Laboratory of Medicinal Chemical Biology, Key Laboratory of Bioactive Materials of Ministry of Education, Nankai University, Tianjin, China

**Keywords:** ALD, roxadustat, fatty liver, HIF-1α, inflammation, oxidative stress

## Abstract

Alcoholic liver disease (ALD) is a worldwide healthcare problem featured by inflammation, reactive oxygen species (ROS), and lipid dysregulation. Roxadustat is used for chronic kidney disease anemia treatment. As a specific inhibitor of prolyl hydroxylase, it can maintain high levels of hypoxia-inducible factor 1α (HIF-1α), through which it can further influence many important pathways, including the three featured in ALD. However, its effects on ALD remain to be elucidated. In this study, we used chronic and acute ALD mouse models to investigate the protective effects of roxadustat *in vivo*. Our results showed that long- and short-term alcohol exposure caused rising activities of serum transaminases, liver lipid accumulation, and morphology changes, which were reversed by roxadustat. Roxadustat-reduced fatty liver was mainly contributed by the reducing sterol-responsive element-binding protein 1c (SREBP1c) pathway, and enhancing β-oxidation through inducing peroxisome proliferator-activated receptor α (PPARα) and carnitine palmitoyltransferase 1A (CPT1A) expression. Long-term alcohol treatment induced the infiltration of monocytes/macrophages to hepatocytes, as well as inflammatory cytokine expression, which were also blocked by roxadustat. Moreover, roxadustat attenuated alcohol caused ROS generation in the liver of those two mouse models mainly by reducing cytochrome P450 2E1 (CYP2E1) and enhancing superoxidase dismutase 1 (SOD1) expression. *In vitro*, we found roxadustat reduced inflammation and lipid accumulation mainly *via* HIF-1α regulation. Taken together, our study demonstrates that activation of HIF-1α can ameliorate ALD, which is contributed by reduced hepatic lipid synthesis, inflammation, and oxidative stress. This study suggested that roxadustat could be a potential drug for ALD treatment.

## Introduction

The alcohol intake-associated disease, that is, alcoholic liver disease (ALD), remains a serious global human health problem. ALD usually starts with hepatic steatosis, and then develops into alcoholic steatohepatitis, cirrhosis, and even hepatocellular carcinoma (Ceni et al., 2014; Kong et al., 2021). Once ALD progresses to steatohepatitis, abstinence from alcohol cannot totally reverse liver damage. Therefore, it is necessary to treat ALD in the stage of hepatic steatosis. The molecular mechanisms of ALD are not well studied, but there is growing evidence that multiple factors are involved in the pathogenesis of ALD, such as oxidative stress, which can promote lipid peroxidation and accelerate fat deposition in the liver, inflammation, as well as dysregulation of gut microbiota (Xu et al., 2017; Meng et al., 2018). The current treatment of ALD is limited to alcohol withdrawal and a few medications, such as polyene phosphatidylcholine, glucocorticoids, and metadoxine. However, the usage of these drugs has some limitations and side effects. Polyene phosphatidylcholine cannot reverse the pathology of ALD (Wang et al., 2019). Glucocorticoids increase the risk of obesity, hypertension, and cardiovascular diseases (Dixon and Bansback, 2012). Metadoxine has the potential to cause diarrhea (Addolorato et al., 2003). Therefore, the development of new drugs for ALD is urgently needed.

Hypoxia-inducible factor (HIF)-1 is a heterodimeric transcription factor consisting of α- and β-subunits that acts as a master regulator of adaptation to hypoxia. Under conditions of oxygen sufficiency, the α-subunit is hydroxylated at its specific proline residues, resulting in rapid degradation. When exposed to hypoxia, the α-subunit is stabilized and translocated to the nucleus to dimerize with HIF-1β, and then to activate its target genes, such as glucose transporters, glycolysis enzymes, and lipid synthases (Kaelin and Ratcliffe, 2008; Rahtu-Korpela et al., 2014). Alcohol exposure increases liver oxygen consumption and subsequently causes hypoxia in the region surrounding the liver lobules (Tsukamoto and Xi, 1989; Arteel et al., 1997). Chronic hypoxia impairs mitochondrial-mediated fatty acid oxidation through the production of reactive oxygen species (ROS), causing mitochondrial dysfunction, which further affects liver lipid synthesis (Lieber, 2004). Reduced oxygen availability initiates the hypoxic response and is a survival mechanism that evolves to enable organisms to cope with low oxygen levels (Kaelin and Ratcliffe, 2008; Koivunen et al., 2016; Gunton, 2020). Roxadustat (FG-4592) can enhance the stabilization of HIF-1α, and is the first small molecule approved for the treatment of renal anemia by promoting the production of erythropoietin and iron utilization (Huang et al., 2020). Some studies have shown that HIF-1 activation can protect against metabolic disorders by reducing serum cholesterol and glucose levels, and improving insulin sensitivity in type 2 diabetic mice (Rahtu-Korpela et al., 2014; Chen et al., 2015). In addition, the development of atherosclerosis can also be attenuated by FG-4592, which is related to the elimination of hepatocyte cholesterol, and thermogenesis (Zhang et al., 2019; Sugahara et al., 2020). However, little has been reported that HIF-1α controls the lipid metabolism in ALD.

Chronic alcohol consumption is a leading cause of ALD. Accumulating evidence indicated that consuming excess alcohol and being overweight synergistically promoted the development of ALD (Lu et al., 2004). Mice fed with the Lieber–DeCarli liquid diet containing ethanol for 8 weeks plus a single binge ethanol feeding (the NIAAA model) could develop ALD, with the characteristics of inflammation and fatty liver, which were wildly used for ALD research (Bertola et al., 2013). Research study has shown that short-term high-fat diet (HFD) feeding could impair glucose tolerance and insulin sensitivity, along with hepatic inflammatory response and liver damage (Chang et al., 2015). Feeding mice with an HFD for 3 days or 8 weeks plus a single gavage of ethanol can induce liver injury by elevating fatty acid accumulation and hepatic neutrophil infiltration. To explore the role of HIF-1α activation on ALD, we used the NIAAA model and HFD plus ethanol mouse model in this study. Along with the construction of ALD mouse models, mice received roxadustat treatment, followed by the determination of ALD development, as well as involved mechanisms.

## Materials and Methods

### Reagents

Roxadustat was provided by Zhejiang Jianfeng Pharmaceutical Co., Ltd. (Jinhua, China). Bovine serum albumin (BSA) was purchased from Sigma Aldrich (Missouri, United States). Hematoxylin and eosin (H&E) staining solution, 4% polyformaldehyde, phosphate buffer saline (PBS), and BCA Protein Assay Kit were purchased from Biosharp (Hefei, China). Total RNApure reagent (Trizol) was purchased from Beijing Zomen Biotechnology Co., Ltd. (Beijing, China). HiScript II Q Select RT SuperMix and AceQ SYBR qPCR Master Mix were purchased from Vazyme (Nanjing, China). The dihydroethidium (DHE) staining kit was purchased from Beyotime (Shanghai, China). A cocktail of protease inhibitors, PMSF, and enhanced chemiluminescence (ECL) kit were purchased from Millipore (Darmstadt, Germany). Bromphenol blue, triton X-100, and sodium dodecyl sulfate (SDS) were purchased from Solarbio (Beijing, China). Mouse anti-fatty acid synthase (FASN) and CD68 monoclonal antibodies were purchased from Santa Cruz Biotechnology (CA, United States). Mouse anti-glyceraldehyde-3-phosphate dehydrogenase (GAPDH), rabbit anti-superoxidase dismutase 1 (SOD1), SOD2, α-Tubulin, peroxisome proliferator-activated receptor α (PPARα), and interleukin-1β (IL-1β) polyclonal antibodies were purchased from Abclonal (Wuhan, China). Rabbit anti-HIF-1α, carnitine palmitoyltransferase 1A (CPT1A), and cytochrome P450 2E1 (CYP2E1) polyclonal antibodies were purchased from Affinity Biosciences (OH, United States). Mouse anti-tumor necrosis factor α (TNF-α), rabbit anti-sterol-responsive element-binding protein 1c (SREBP1c), carbohydrate response element-binding protein α (ChREBPα) polyclonal antibodies, HRP-conjugated goat anti-rabbit IgG (H + L), and mouse IgG (H + L) were purchased from Proteintech Group Inc. (IL, United States). All other chemical reagents were analytical grade.

### Cell Culture

HepG2 and RAW264.7 cells were purchased from ATCC (VA, United States), and cultured in complete MEM or 1640 medium containing 10% fetal bovine serum (FBS, AusGeneX, Australia) and 50 μg/ml streptomycin/penicillin (UT, United States), in a humidified incubator with 5% CO_2_ at 37°C. Before treatment, cells were incubated in a serum-free medium.

### siRNA Transfection

*Homo* HIF-1α siRNA and the corresponding scrambled siRNA were purchased from RiboBio Biotechnology (Guangzhou, China). HepG2 cells were cultured in a 6-well plate at a density of 5 × 10^5^ cells/well in a serum-free Opti-MEM. HIF-1α or control siRNA (40 nM/well) were transfected into cells using Lipofectamine RNAiMAX Transfection Reagent (Invitrogen, CA, United States). After 24 h transfection, HepG2 cells received indicated treatment (Wang et al., 2020).

### *In Vivo* Studies

The eight-week-old male C57BL/6J mice were purchased from GemPharmatech (Nanjing, China). Mice were maintained in a chamber with constant temperature (22 ± 2°C) and humidity (55 ± 2%) for a 12-h light/dark cycle.

The chronic ALD mouse model was constructed as described (Bertola et al., 2013). In brief, mice were divided into four groups (8 mice/group); all mice were fed with the Lieber–DeCarli control diet for the first 5 days. Then, mice in control groups were fed with the Lieber–DeCarli control diet (ethanol free) for 8 weeks plus intragastric (i.g.) administration of a single maltose dextrin solution (9 g/kg body weight, equal calorie to ethanol); mice in ALD groups were fed with the Lieber–DeCarli diet (contain 5% ethanol) for 8 weeks plus i.g. administration of single binge ethanol (5 g/kg body weight). Mice were euthanized after 9 h of the single binge ethanol or maltose solution administration.

The acute ALD mouse model was constructed as follows (Chang et al., 2015): mice in the control groups were fed normal chow for 3 days, and then received i.g. administration of maltose dextrin solution (9 g/kg body weight). In model groups, mice were fed an HFD (60% kcal; CAT#D12492) for 3 days and received i.g. administration of 31.25% (vol/vol) ethanol solution (5 g/kg body weight) on the last day. All mice were euthanized after 9 h of ethanol or maltose dextrin solution administration, followed by a collection of blood and tissue samples.

To determine the role of roxadustat in ALD, mice in the vehicle group received intraperitoneal (i.p.) injection of PBS every day; mice in roxadustat groups received i.p. injection of roxadustat solution (10 mg/kg body weight or 25 mg/kg body weight for chronic or acute ALD mouse model, respectively) every day. The selection for doses of roxadustat is described as follows: previous studies used a serious range of roxadustat for *in vivo* experiments, mainly from 10 to 60 mg/kg body weight (Beck et al., 2017; Deguchi et al., 2020; Kabei et al., 2020). To verify if different doses of roxadustat have protective effects in acute and chronic ALD mouse models, we chose a low dose of roxadustat for the chronic model, while a middle dose for the acute model, which is based on the standard of animal ethics that use as fewer animals as possible in *in vivo* experiment.

### Western Blot and Immunohistochemical Staining

After treatment, cells or 30 mg liver tissues were lysed or grated with lysis buffer. The BCA Protein Assay Kit was used to determine protein concentration. The same amount of protein (60 μg) from each sample was used to determine the protein expression of ACC1, SREBP1c, FASN, IL-1β, PPARα, CPT1A, CYP2E1, TNF-α, ChREBPα, HIF-1α, GAPDH, and α-Tubulin using Western blot (Yin et al., 2020). The signals were detected by ChemiScope 3000 mini (Qinxiang, Shanghai, China), and the band density was quantified using Photoshop software.

Liver CD68 expression was determined using immunohistochemical staining. Images were obtained using a ZEISS Scope A1 fluorescence microscope, and quantity analysis of CD68 positive cells was determined by Photoshop software.

### Quantitative Real-Time PCR (qRT-PCR)

After treatment, Trizol was used to extract total RNA from 20 mg liver tissues or RAW264.7 cells. cDNA was synthesized with 1 μg RNA from each sample with HiScript II Q Select RT SuperMix (gDNA wiper). RT-PCR was applied with the primers listed in Table 1. mRNA expression was normalized by β-actin mRNA in the corresponding samples.

**TABLE 1:** q-RT-PCR primer sequences.

Gene	Forward	Backward
Mus ACC1	GCC​ATT​GGT​ATT​GGG​GCT​TAC	CCC​GAC​CAA​GGA​CTT​TGT​TG
Mus β-actin	ATG​GAG​GGG​AAT​ACA​GCC​C	TTC​TTT​GCA​GCT​CCT​TCG​TT
Mus ChREBPα	GTC​CCC​GCA​GGA​TAC​AGT​TT	TTG​TTG​TCT​ACA​CGA​CCC​CG
Mus DGAT1	GGT​GCC​CTG​ACA​GAG​CAG​AT	CAG​TAA​GGC​CAC​AGC​TGC​TG
Mus FASN	CTG​CGA​TGA​AGA​GCA​TGG​TTT	CCA​TAG​GCG​ATT​TCT​GGG​AC
Mus IL-1β	GAC​CTT​CCA​GGA​TGA​GGA​CA	AGC​TCA​TAT​GGG​TCC​GAC​AG
Mus IL-6	GAG​GAT​ACC​ACT​CCC​AAC​AGA​CC	AAG​TGC​ATC​ATC​GTT​GTT​CAT​ACA
Mus PPARα	AGT​TCG​GGA​ACA​AGA​CGT​TG	CAG​TGG​GGA​GAG​AGG​ACA​GA

ACC1, acetyl-CoA carboxylase 1; ChREBPα, carbohydrate response element-binding protein α; DGAT, acyl-CoA: diacylglycerol acyltransferase; FASN, fatty acid synthase; IL-1β/6, interleukin-1β or 6; PPARα, peroxisome proliferator-activated receptor α.

### H&E, Oil Red O, and DHE Staining

A piece of the liver was fixed in 4% paraformaldehyde overnight, and then dehydrated with an auto dehydrator (Leica, Wetzlar, Germany). After being embedded in paraffin, the tissue samples were cut into 5 μm sections, and then conducted with H&E staining. Frozen liver tissues embedded in OCT were cut into 5 μm sections for the determination of lipid accumulation or ROS levels by Oil Red O or DHE staining, respectively (Wang et al., 2021). Images were obtained using a ZEISS Scope A1 fluorescence microscope. DHE fluorescence intensity was quantified using ImageJ software.

### Statistical Analysis

All data were generated from at least three independent experiments. GraphPad Prism 8.0 was used for data statistical analysis. All data were shown as means ± SEM. Data were analyzed by one-way ANOVA followed by Bartlett’s test, and the difference was considered significant at *p* < 0.05.

## Results

### Roxadustat Inhibits the Development of Chronic Alcoholic Liver Disease

**FIGURE 1:**
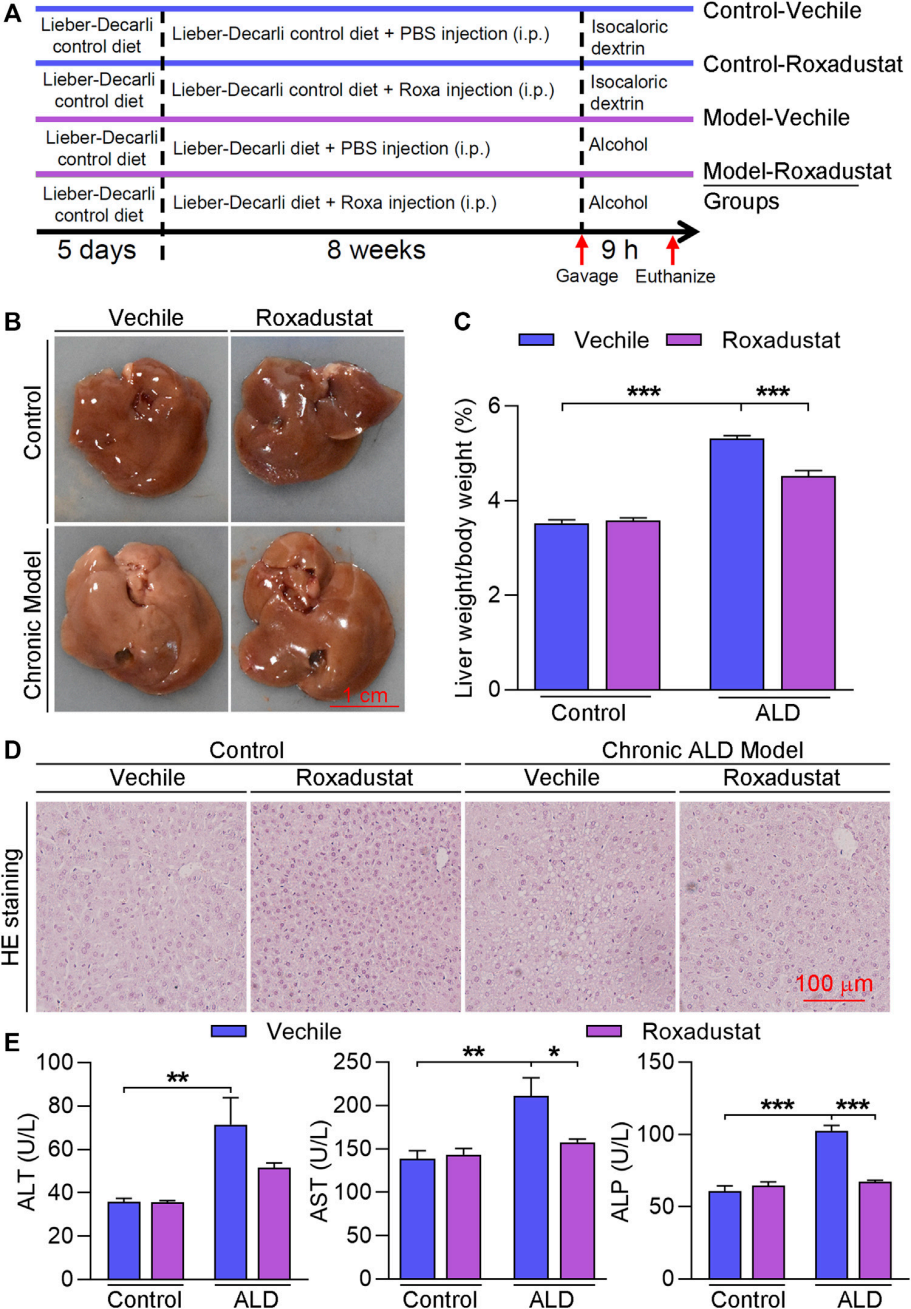
Roxadustat inhibits the development of chronic ALD. **(A)** (experimental design): C57BL/6J mice in four groups (8 mice/group) received the following treatment: control groups: fed with the Lieber–DeCarli control diet plus a single gavage of maltose dextrin solution; model groups: fed with Lieber–DeCarli control diet for 5 days, and then with the Lieber–DeCarli diet for 8 weeks plus a single gavage of ethanol (5 g/kg body weight). Mice in vehicle groups received i.p. injection of PBS; mice in roxadustat groups received i.p. injection of roxadustat solution (10 mg/kg body weight) for 8 weeks daily. Mice were sacrificed after 9 h of maltose dextrin or ethanol gavage, blood, and liver tissues were collected; **(B)** liver from each mouse was photographed, and the representative photographs are presented; **(C)** ratio of liver weight to body weight was calculated; **(D)** liver paraffin sections were conducted with H&E staining; **(E)** serum was used to determine ALT, AST, and ALP activities using an automatic biochemical analyzer. *, *p* < 0.05; **, *p* < 0.01; ***, *p* < 0.001 (n ≥ 5).

To investigate the role of roxadustat on ALD, we first constructed a chronic ALD mouse model by feeding the Lieber–DeCarli liquid diet plus a single alcohol gavage, and mice received roxadustat treatment for 8 weeks (Figure 1A). During the experiment, we monitored the pathology changes in the liver with an ultrasound scanner (Mathiesen et al., 2002; Pandit et al., 2019). The B-mode ultrasound images indicated the Lieber–DeCarli liquid diet caused hepatic steatosis after 2 weeks of feeding, evidenced by enhanced brightness. In contrast, lipid accumulation was attenuated along with roxadustat treatment (Supplementary Figure S1). In addition, we found long-term exposure to alcohol resulted in an overall larger and whiter liver, as well as an elevated ratio of liver weight to body weight, which was improved by roxadustat treatment (Figures 1B,C). Consistent with morphological changes in the liver, H&E staining revealed significant pathological morphological changes in chronic ALD mouse liver (Figure 1D). However, roxadustat improved the damage on liver tissues. Serum transaminase activities are indicators of liver damage, which can be produced by injured hepatocytes (Marin et al., 2017). Our results showed that serum ALT, AST, and ALP activities were enhanced in chronic ALD mouse serum while being attenuated by roxadustat, especially the AST and ALP activities (Figure 1E). The aforementioned results indicated that roxadustat inhibits the development of chronic ALD.

### Roxadustat Improves Liver Lipid Accumulation by Inhibiting Triglyceride Synthesis-Related Gene Expression and Inducing PPARα Levels in Chronic Alcoholic Liver Disease Mice

**FIGURE 2:**
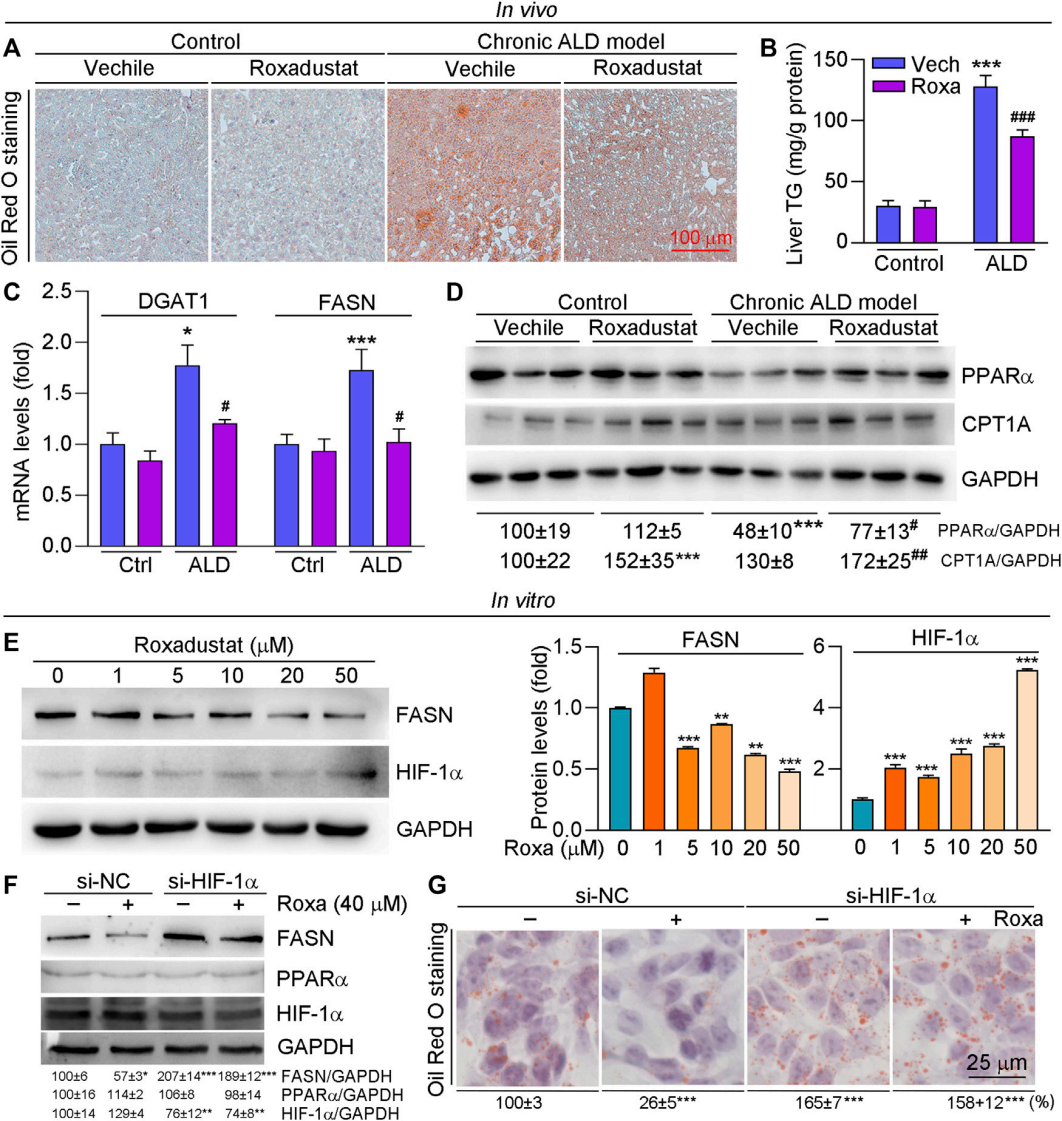
Roxadustat attenuates lipid accumulation by increasing PPARα protein expression in the liver and reducing FASN levels in HepG2 cells. **(A–D)** Liver samples were collected from mice in Figure 1 and used for the following experiments. Liver frozen sections were stained with Oil Red O staining **(A)**; triglyceride content was determined using an assay kit **(B)**; mRNA expression of DGAT1 and FASN was determined by qRT-PCR **(C)**; protein expression of PPARα and CPT1A was determined by Western blot with quantitative analysis of band density **(D)**; **(E)** HepG2 cells were treated with roxadustat at indicated concentrations for 24 h. Protein expression of FASN and HIF-1α was determined by Western blot with quantitative analysis of band density (right panels); **(F,G)** HepG2 cells were transfected with scrambled siRNA (si-NC) or HIF-1α siRNA (si-HIF-1α) for 24 h, and then treated with roxadustat for 24 h. Protein expression of FASN, PPARα, and HIF-1α was determined by Western blot with quantitative analysis of band density (F); lipid accumulation was determined by Oil Red O staining with quantitative analysis (G). *, *p* < 0.05; **, *p* < 0.01; ***, *p* < 0.001 vs ctrl; ^#^ *p* < 0.05, ^###^, *p* < 0.001 vs ALD group (n ≥ 5); Roxa: roxadustat.

The results of B-mode ultrasound and H&E staining indicated that roxadustat can improve Lieber–DeCarli liquid diet-induced hepatic steatosis. We further conducted Oil Red O staining and found that lipid accumulation was enhanced in the chronic ALD group while being alleviated by roxadustat (Figure 2A). Compared to the model group, liver triglyceride levels were also attenuated by roxadustat treatment (Figure 2B). DGAT and FASN are key enzymes that catalyze the final reaction of triglyceride synthesis (Chitraju et al., 2017). Our results showed that DGAT and FASN mRNA levels were increased under the mediation of alcohol (Figure 2C). However, roxadustat treatment reduced alcohol-enhanced DGAT1 and FASN expression. PPARα is a nuclear receptor that regulates the expression of various genes involved in mitochondrial β-oxidation (You and Arteel, 2019). Alcohol intake affects mitochondrial oxidation and inhibits PPARα signaling. As shown in Figure 2D, PPARα protein expression was reduced in ALD mouse liver, which was enhanced in the roxadustat treatment group. CPT1A is a classical target gene of PPARα, a key regulator for fatty acid β-oxidation (Rakhshandehroo et al., 2010). Consistent with the results of PPARα, we also found that roxadustat treatment enhanced CPT1A protein levels (Figure 2D).

To further investigate the mechanism of roxadustat on lipid accumulation, we treated HepG2 cells with roxadustat and found that roxadustat reduced FASN protein expression in a dose-dependent manner (Figure 2E). Furthermore, we transfected cells with HIF-1α siRNA to knockdown HIF-1α levels. As shown in Figure 2F, roxadustat inhibited FASN expression in siNC HepG2 cells while having little effect on siHIF-1α HepG2 cells, indicating roxadustat regulates FASN levels depending on HIF-1α expression. In addition, Oil Red O staining results showed that roxadustat inhibited lipid accumulation in control cells, but not in HIF-1α knockdown cells (Figure 2G). In contrast to the *in vivo* results, we found roxadustat had little effect on PPARα protein levels neither in control cells nor in HIF-1α knockdown cells (Figure 2F). Taken together, the abovementioned results demonstrated that roxadustat inhibits lipid accumulation both *in vivo* and *in vitro*.

### Roxadustat Inhibits Inflammatory Response in Chronic Alcoholic Liver Disease Mice

**FIGURE 3:**
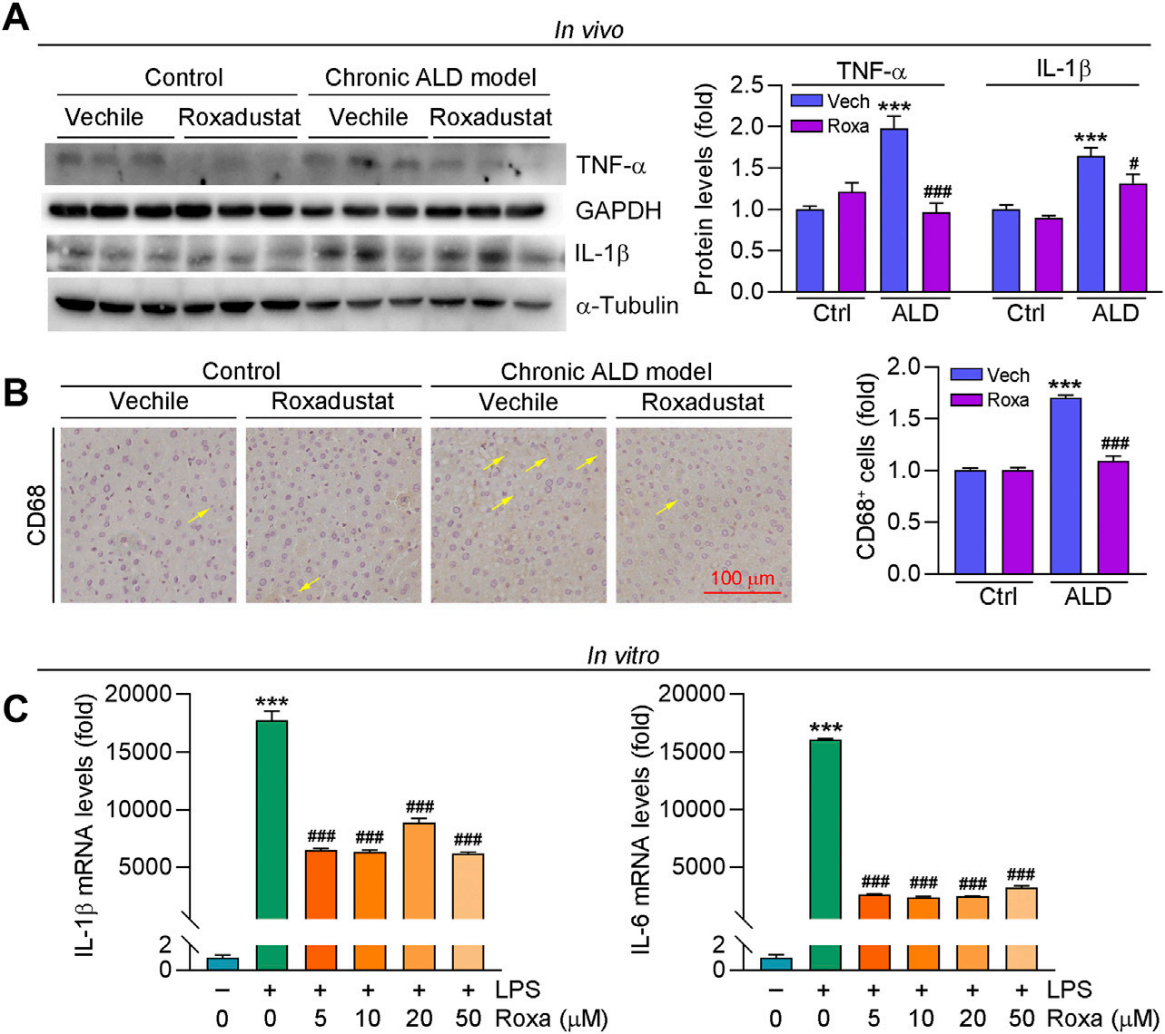
Roxadustat reduces inflammation by reducing inflammatory cytokines both *in vivo* and *in vitro*. **(A,B)** Liver samples were collected from mice in Figure 1, protein expression of TNF-α and IL-1β were determined using Western blot with quantitative analysis of band density **(A)**, CD68 protein expression was determined using immunohistochemical staining with quantitative analysis **(B)**. **(C)** RAW264.7 cells were pretreated with indicated concentrations of roxadustat for 2 h, and then co-treated with LPS (1 μg/ml) for 24 h. mRNA levels of IL-1β and IL-6 were determined by qRT-PCR. ***, *p* < 0.001 vs ctrl; ^#^, *p* < 0.05, ^###^, *p* < 0.001 vs ALD group or LPS-treated group (n ≥ 3); Roxa: roxadustat.

Alcohol and its metabolic derivatives can act as harmful stimuli to the body, leading to an inflammatory response. Inflammation maintains the homeostasis of the body, but can also cause collateral damage to normal tissues (Xu et al., 2017). Alcohol impairs intestinal barrier function and increases LPS flux to the portal vein. Excess LPS binds to toll-like receptor four to activate macrophages, causing inflammatory cytokine secretion (Xu et al., 2017). In this study, we found pro-inflammatory cytokines expression, such as IL-1β and TNF-α, was increased in liver tissues of ALD mice, which was significantly inhibited by roxadustat (Figure 3A). In addition, we conducted immunohistochemical staining with CD68 antibody (a marker for monocyte) and found that levels of CD68^+^ cells were enhanced in ALD mouse liver. Consistent with the results of inflammatory cytokines, roxadustat reduced the infiltration of monocytes (Figure 3B). Furthermore, we treated RAW264.7 cells with LPS to induce inflammation in the presence or absence of roxadustat. As shown in Figure 3C, LPS-induced IL-1β and IL-6 mRNA levels were largely attenuated by roxadustat treatment. The above results suggest that roxadustat inhibits inflammatory response both *in vivo* and *in vitro*.

### Roxadustat Inhibits the Development of Acute Alcoholic Liver Disease

**FIGURE 4:**
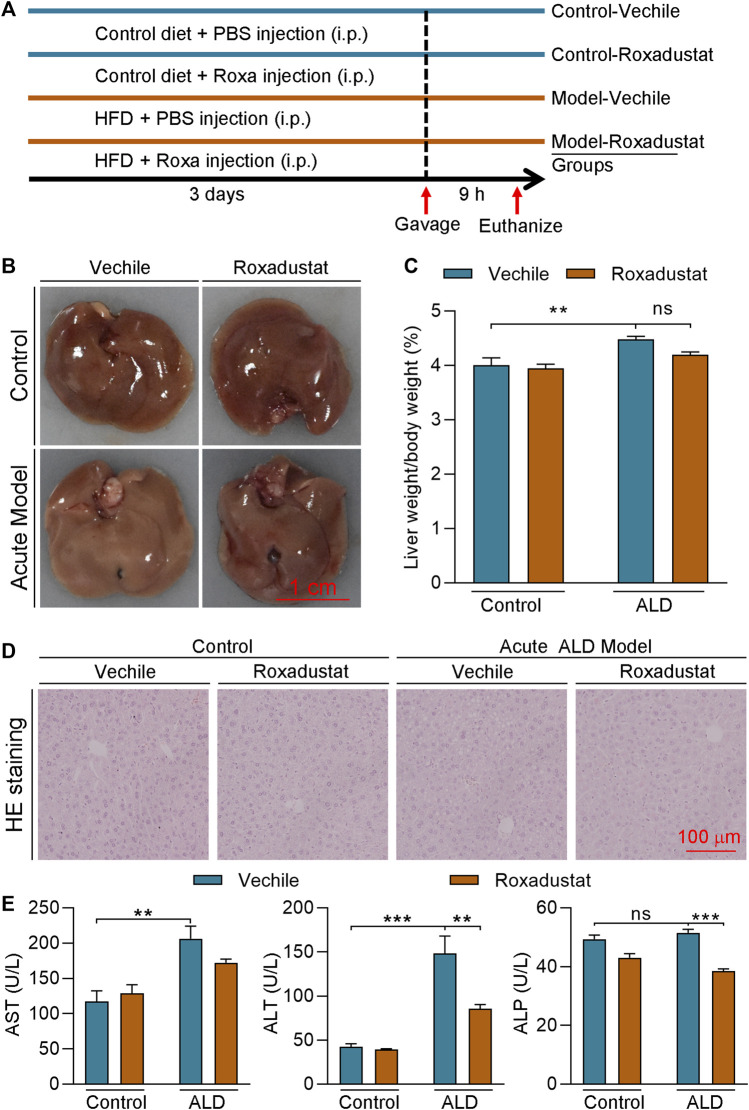
Roxadustat inhibits the development of acute ALD **(A)** (experimental design): C57BL/6J mice in four groups (6 mice/group) received the following treatment: control groups: fed with normal chow for 3 days plus a single gavage of maltose dextrin solution; model groups: fed with HFD for 3 days plus i.g. administration of 31.25% (vol/vol) ethanol solution (5 g/kg body weight) on the last day. Mice in the vehicle groups received i.p. injection of PBS; mice in roxadustat groups received i.p. injection of roxadustat solution (25 mg/kg body weight) daily. Mice were sacrificed after 9 h of maltose dextrin or ethanol gavage, blood, and liver tissues were collected; **(B)** liver from each mouse was photographed, and the representative photographs were presented; **(C)** and the ratio of liver weight to body weight was calculated; **(D)** liver paraffin sections were stained with H&E staining; and **(E)** serum was used to determine ALT, AST, and ALP activities by an automatic biochemical analyzer. **, *p* < 0.01; ***, *p* < 0.001; ns: not significantly different (n ≥ 5).

To further investigate if roxadustat has protective effect on HFD plus acute alcohol-caused liver injury, we conducted an acute ALD mouse model by feeding mice an HFD for 3 days plus a single gavage of ethanol (Figure 4A). After 9 h of ethanol treatment, the mice were sacrificed. Mouse liver of the model group showed a tendency to be much whiter and larger, with an enhanced ratio of liver weight to body weight (Figures 4B,C). In contrast, roxadustat attenuated acute alcohol-caused changes in the liver and slightly decreased liver weight/body weight. Moreover, fat vacuoles were found in acute ALD mouse liver, which were improved by roxadustat (Figure 4D). Consistent with the results of the chronic ALD mouse model, we showed acute alcohol also enhanced serum ALT, AST, and ALP activities (Figure 4E). However, AST and ALP activities were greatly inhibited by roxadustat treatment, indicating the hepatoprotective function of roxadustat.

### Roxadustat Attenuates Lipid Accumulation in Acute Alcoholic Liver Disease Mouse Liver by Regulating Hepatic Lipid Synthesis

**FIGURE 5:**
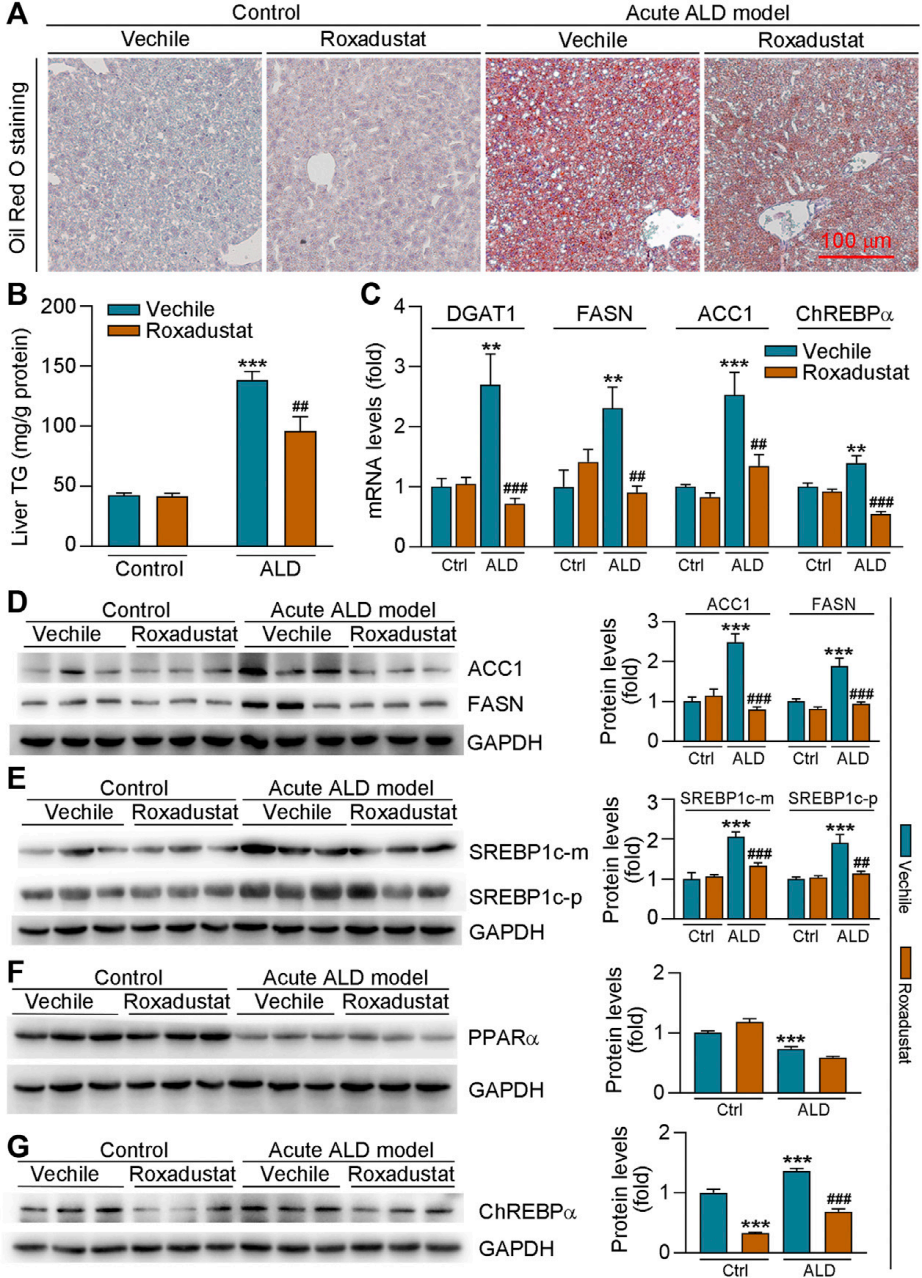
Roxadustat reduces lipid accumulation in acute ALD mouse liver by decreasing fatty acid synthesis-related gene expression. Liver samples were collected from mice in Figure 4, and liver frozen sections were conducted with Oil Red O staining **(A)**; mouse liver triglyceride content was measured with an assay kit **(B)**; mRNA levels of DGAT1, FASN, ACC1, and ChREBPα were determined by qRT-PCR **(C)**; **(D-G)** protein expression of ACC1, FASN **(D)**, precursor (p), or mature (m) form of SREBP1c **(E)**, PPARα **(F)**, and ChREBPα **(G)** was determined by Western blot with quantitative analysis of band density (right panels). **, *p* < 0.01; ***, *p* < 0.001 vs ctrl group; ^##^, *p* < 0.01, ^###^, *p* < 0.001 vs ALD group (n ≥ 3).

The results of H&E staining in Figure 4D showed that roxadustat can also inhibit acute alcohol-induced lipid accumulation in the liver. We further conducted Oil Red O staining of liver frozen sections and found that roxadustat treatment significantly reduced lipid accumulation in acute ALD model mouse liver (Figure 5A). The results of liver triglyceride levels further confirmed that lipid levels were attenuated by roxadustat (Figure 5B). The abovementioned results indicated that roxadustat can inhibit acute alcohol-induced hepatic lipid accumulation. The liver generates fatty acids from non-lipid precursors *via de novo* lipogenesis. Multiple enzymes participate in this process; ACC-1 converts acetyl coenzyme A to malonyl coenzyme A and FASN synthesizes saturated fatty acids from malonyl coenzyme A (Huang et al., 2010). Compared to the control group, we found mRNA levels of DGAT1, FASN, and ACC1, and protein levels of ACC1 and FASN were induced in acute ALD mouse liver. However, the expression of the aforementioned lipogenesis-related genes was inhibited by roxadustat (Figures 5C,D). SREBP1c, a key transcription factor, is a master regulator of lipogenesis by activating genes related to fatty acid and triglyceride synthesis (Li et al., 2011). Our results showed that acute alcohol-induced both precursor and mature forms of SREBP1c protein expression were reduced by roxadustat treatment (Figure 5E). However, we found that roxadustat had little effect on acute alcohol-reduced PPARα protein expression (Figure 5F), which is consistent with the results of Figure 2D. ChREBPα is emerging as a critical driver of the lipid metabolism (Wang et al., 2015; Sanchez-Gurmaches et al., 2018). Our results indicated that ChREBPα mRNA and protein expression were enhanced in acute ALD mouse liver, which was reduced by roxadustat treatment (Figures 5C,G). Taken together, the above results suggested that roxadustat can attenuate the development of acute ALD through downregulation of lipid synthesis-related genes expression.

### Roxadustat Improves Oxidative Stress in Both Chronic and Acute Alcoholic Liver Disease Mouse Models by Reducing Cytochrome P450 2E1 Expression and Enhancing Superoxidase Dismutase 1 Levels

**FIGURE 6:**
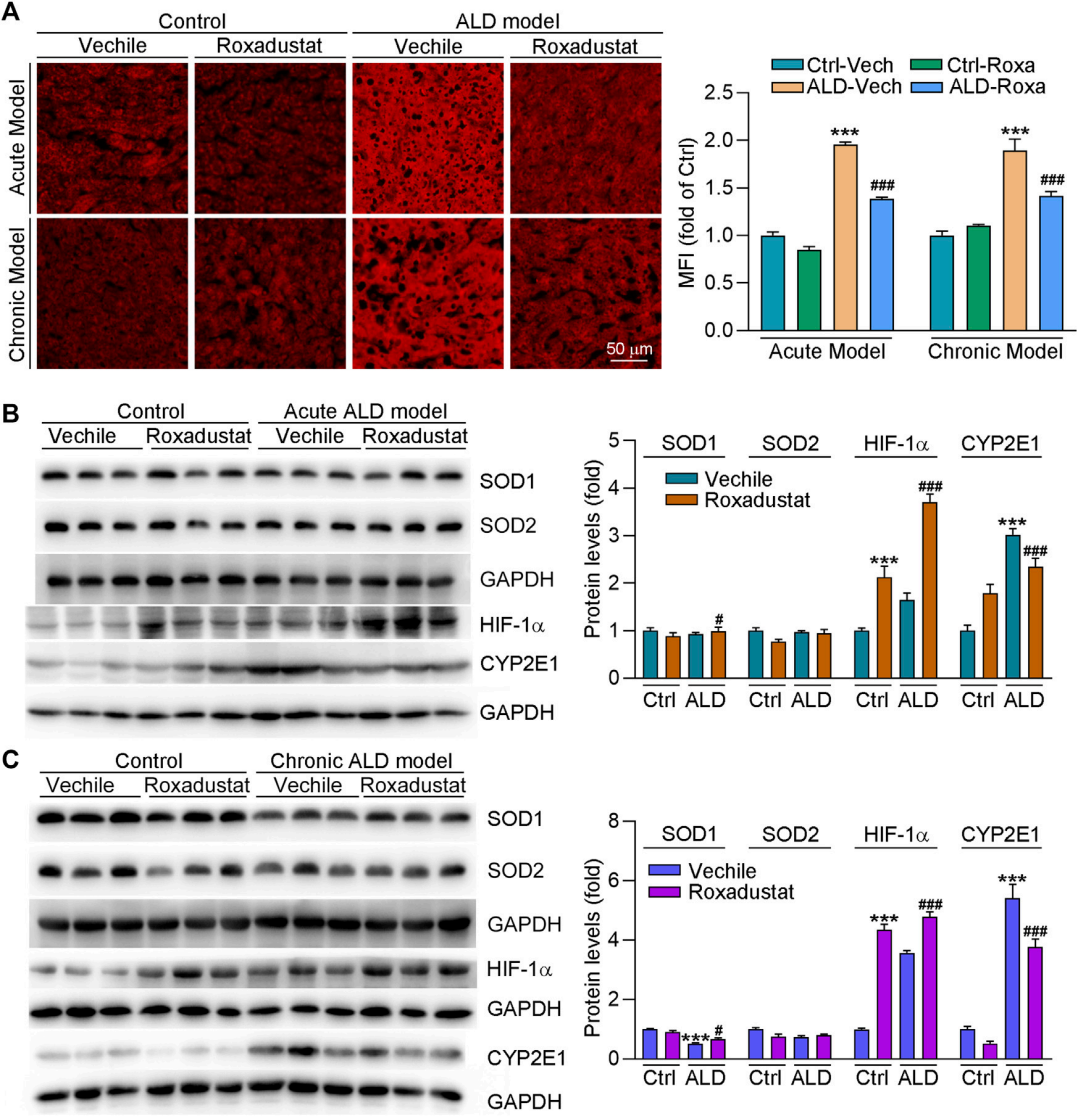
Roxadustat reduces oxidative stress in mouse liver by enhancing SOD1 and decreasing CYP2E1 expression. **(A)** Superoxide in the liver was determined by DHE staining; **(B**,**C)** total protein extracted from the liver of acute ALD mouse **(B)** and chronic ALD mouse **(C)** was used to determine the protein expression of SOD1, SOD2, HIF-1α, and CYP2E1 using Western blot with quantitative analysis of band density (right panels). ***, *p* < 0.001 vs ctrl group; ^#^, *p* < 0.05, ^###^, *p* < 0.001 vs ALD group (n ≥ 3).

The alcohol metabolism generates large amounts of free radicals and ROS, which can cause oxidative stress and mitochondrial damage to lead further damage and apoptosis of hepatocytes. To determine if roxadustat can regulate oxidative stress in ALD mouse models, we conducted DHE staining of liver sections. As shown in Figure 6A, we found that ROS levels were enhanced in both chronic and acute ALD mouse liver. However, roxadustat decreased ROS accumulation in liver tissues, evidenced by reduced density of red fluorescence. CYP2E1 is an alcohol-inducible enzyme that contributes to ethanol metabolism. It can cause oxidative stress, depletion of the antioxidant system, and liver damage due to massive rupture of hepatocyte mitochondrial membranes (Albano, 2008). We found protein expression of CYP2E1 was largely enhanced in ALD mouse liver. Conversely, roxadustat reduced alcohol-enhanced CYP2E1 levels in those two mouse models with HIF-1α activation (Figures 6B,C). SOD1 and SOD2 are critical antioxidant enzymes. Our results showed that roxadustat had little effect on SOD2 protein expression. However, inhibited SOD1 levels were enhanced by roxadustat in both acute and chronic ALD mouse liver tissues (Figures 6B,C). The aforementioned results showed that roxadustat ameliorates alcohol-induced ROS accumulation by decreasing CYP2E1 and enhancing SOD1 expression.

**FIGURE 7:**
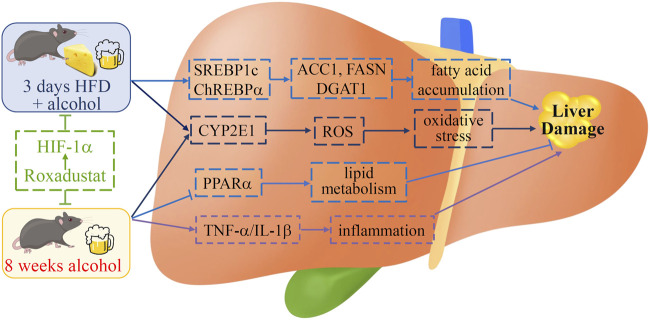
Schematic diagram of the role of roxadustat in ALD. Roxadustat reduces long- and short-term alcohol-induced liver damage. Activation of HIF-1α ameliorates fatty acid accumulation by regulating SERBP1c and ChREBPα in acute ALD mice while improving the lipid metabolism through the PPARα pathway in chronic ALD mice. Roxadustat blocks the expression of long-term alcohol treatment-induced inflammatory cytokines TNF-α and IL-1β. Meanwhile, roxadustat reduces oxidative stress by reducing hepatic CYP2E1 to keep ROS at a low level in those two mouse models. We indicate that roxadustat may be a potential drug for ALD.

## Discussion

Roxadustat is a clinical drug used for treating anemia in chronic kidney disease patients. In this study, we used chronic and acute ALD mouse models to explore the protective role of roxadustat in liver diseases. Our study demonstrates that roxadustat inhibits the development of ALD, which was evidenced by reducing serum aminotransferase activities, fatty liver, inflammation, and ROS levels. Mechanistically, roxadustat reduced the expression of fatty acid synthesis-related genes expression, including SREBP1c, ChREBPα, FASN, and ACC1, and enhanced the β-oxidation pathway by promoting the expression of PPARα and CPT1A (Figures 2, 5). At the same time, roxadustat decreased the expression of inflammatory factors IL-6, IL-1β, and TNF-α in the liver, and inhibited macrophage/monocyte migration to ameliorate long-term alcohol-induced inflammation (Figure 3). Moreover, we indicated that roxadustat ameliorated oxidative stress by inhibiting CYP2E1 and promoting SOD1 expression in the liver (Figure 6).

Chronic alcohol abuse can not only induce liver damage but also impair renal tubular function (Labib et al., 1989). A nationwide database analysis indicated that the incidence of chronic kidney disease is positively related to alcohol use disorder (Pan et al., 2018). Previous studies have shown that alcohol can reduce renal function and interstitial edema in rat kidneys (Van Thiel et al., 1977; Sönmez et al., 2012; Varga et al., 2017). The liver is the dominant organ for the alcohol metabolism and the target organ of toxicity. Alcohol consumption enhances ROS levels in the liver, as well as other tissues, thereby causing serious damage, such as fibrosis, ferroptosis, and DNA damage. Anemia can be caused by both chronic kidney and liver diseases with the feature of decreased hemoglobin and circulating erythrocytes, which is related to inadequate production of erythropoietin in the kidney, accumulation of inflammation, and deficiency of iron (Gonzalez-Casas et al., 2009; Koury and Haase, 2015). HIF activation promotes erythropoietin transcription in both the kidney and liver to alleviate anemia (Gonzalez-Casas et al., 2009; Koury and Haase, 2015). Moreover, anemia is a frequent complication of advanced liver disease (Gkamprela et al., 2017). Therefore, amelioration of anemia benefits chronic kidney and liver diseases.

The early accumulation of triglycerides in hepatocytes can be regulated by several pathways. In the process of ALD, alcohol reduces mitochondrial fatty acid β-oxidation by increasing the level of NADH/NAD^+^ in hepatocytes, which leads to steatosis (Baraona and Lieber, 1979). Alcohol consumption can also upregulate hepatic SREBP1c expression, as well as target lipogenic-related genes, to enhance fatty acid synthesis (Galli et al., 2001). Both short- and long-term treatment of HFD promote hepatic steatosis (Wiedemann et al., 2013), indicating alcohol plus HFD may cause severe liver damage. PPARα is inhibited by long-term treatment of alcohol (Fischer et al., 2003). Previous studies have proven fenofibrate (a PPARα agonist) treatment reverses ethanol-induced liver steatosis by stimulating the β-oxidation pathway (Xu et al., 2021). Some evidence has shown that alcohol increases fatty acid synthesis by increasing the ChREBP activity (Dentin et al., 2005). Our results showed that roxadustat reduced lipid accumulation by increasing PPARα and CPT1A expression and reducing DGAT1 and FASN levels in chronic ALD mouse livers (Figures 2C,D). In contrast to chronic ALD mouse, roxadustat had little effect on PPARα expression in acute ALD mouse liver or HepG2 cells (Figures 2F, 5F), indicating PPARα is not a direct target gene of HIF-1α. On the other hand, we found that roxadustat inhibited fatty acid accumulation by reducing SREBP1c, ChREBPα, DGAT1, ACC1, and FASN expression in the acute ALD mouse liver (Figure 5).

Inflammation plays an important role in the pathogenesis of ALD. Immune cells are the main sources of pro-inflammatory cytokines and chemokines, which lead to the deterioration of ALD. Hepatocytes and liver non-parenchymal cells can also produce pro-inflammatory cytokines. It has been demonstrated that high level of TNF-α in serum is associated with the pathophysiology of alcoholic hepatitis patients (Tilg et al., 2003). Short- or long-term HFD feeding plus acute alcohol binge synergistically induce acute liver injury by enhancing the expression of hepatic chemokine (C-X-C motif) ligand 1 and promoting the infiltration of hepatic neutrophil (Chang et al., 2015). Previous research has proven that stabilization of HIF-1α exerts an anti-inflammatory effect by inhibiting the expression of pro-inflammatory cytokines in murine colitis (Keely et al., 2014). Our results confirmed that alcohol-induced monocyte/macrophage recruitment in mouse liver was reduced by roxadustat (Figure 3B). In addition, roxadustat inhibited alcohol or LPS-enhanced inflammatory cytokines in mouse liver or RAW264.7 cells (Figures 3A,C). Our results showed that roxadustat can also inhibit inflammation in ALD.

Alcohol is oxidized in hepatocytes by ethanol dehydrogenase to acetaldehyde, and then metabolized to acetic acid by acetaldehyde dehydrogenase. Alcohol and its metabolites have toxic, neurodegenerative, or cancerogenic properties (Correa et al., 2003; Quertemont and Didone, 2006; Nieminen and Salaspuro, 2018). ROS elevation is closely associated with the pathology of ALD, and high levels of ROS damage cell structure and lead to cell death by oxidizing nucleic acids, proteins, and lipids (Mittler, 2002). CYP2E1 is an enzyme for metabolizing alcohol to acetaldehyde, and can be induced by the alcohol metabolism, which leads to liver injury and ROS production (Lieber et al., 1970). Ethanol induced the accumulation of macrovesicular fat, and liver triglyceride was blocked in CYP2E1 knockout mice. Compared to wild-type mice, oxidative stress and lipid peroxidation were also reduced in CYP2E1 knockout mice. In contrast, restored CYP2E1 expression by adenovirus in CYP2E1 knockout mice induced fat accumulation in the liver (Lu et al., 2008). In addition to alcohol, HFD can also induce oxidative stress (Matsuzawa-Nagata et al., 2008). Studies have shown that mitochondrial HIF-1α can protect against hypoxia or H_2_O_2_ caused cell apoptosis by reducing oxidative stress (Li et al., 2019). In diabetic rats, a carbohydrate energy-restricted diet attenuates renal damage by upregulating HIF-1α levels to reduce oxidative stress. In addition, HIF-1α activation can protect against doxorubicin-induced cardiotoxicity by inhibiting inflammation and oxidative stress (Long et al., 2020). In this study, we showed that liver ROS generation was attenuated by roxadustat both in acute and chronic ALD mouse models. Our results indicated that roxadustat enhanced SOD1 expression while having little effects on SOD2 levels. However, CYP2E1 expression was largely reduced by roxadustat, indicating roxadustat regulates alcohol-induced liver ROS levels mainly by inhibiting CYP2E1 and enhancing SOD1 expression (Figure 6).

In this study, we used two ALD mouse models to explore the protective role of roxadustat on liver injury. In the chronic model, enhanced inflammatory cytokines and infiltration of monocytes/macrophages were almost blocked by roxadustat, indicating the anti-inflammatory properties of roxadustat. Ethanol or ethanol plus HFD caused fatty liver was largely attenuated by roxadustat. Regarding molecular mechanisms, the anti-ALD effects of roxadustat are contributed by decreased fatty acid accumulation, enhanced β-oxidation, reduced inflammation, and oxidative stress (Figure 7). In conclusion, our results demonstrate that HIF-1α activation provides protection against ALD in both chronic and acute mouse models, and reveal the potential of roxadustat for ALD treatment.

## Data Availability

The original contributions presented in the study are included in the article/Supplementary Material, further inquiries can be directed to the corresponding author.

## Abbreviations

ACC1, acetyl-CoA carboxylase 1; ALD, alcoholic liver disease; ALP, alkaline phosphatase; ALT, alanine aminotransferase; AST, aspartate aminotransferase; ChREBPα, carbohydrate response element-binding protein α; CPT1A, carnitine palmitoyltransferase 1A; CYP2E1, cytochrome P450 2E1; DGAT, diacylglycerol acyltransferase; FASN, fatty acid synthase; GAPDH, glyceraldehyde-3-phosphate dehydrogenase; HFD, high-fat diet; HIF-1α, hypoxia-inducible factor-1α; IL-1β/6, interleukin-1β or 6; PPARα, peroxisome proliferator-activated receptor α; ROS, reactive oxygen species; SOD1/2, superoxidase dismutase 1 or 2; SREBP1c, sterol-responsive element-binding protein 1c; TNF-α, tumor necrosis factor α.
