# Supplementary material for: Roxadustat, a Hypoxia-Inducible Factor 1α Activator, Attenuates Both Long- and Short-Term Alcohol-Induced Alcoholic Liver Disease
Source: Front Pharmacol. 2022 May 10;13:895710. doi: 10.3389/fphar.2022.895710 (PMC9127324; doi:10.3389/fphar.2022.895710)
Supplement: Supplementary file 1 [file DataSheet2.docx]

Roxadustat, a hypoxia-inducible factor 1α activator, attenuates both long- and short-term alcohol induced alcoholic liver disease

Yongyao Gao^1,†^, Xiaomeng Jiang^2^, Daigang Yang^1,†^, Wentong Guo^1^, Dandan Wang^3^, Ke Gong^1^, Ying Peng^1^, Hong Jiang^2^, Cunyuan Shi^2^, Yajun Duan^1^, Yuanli Chen^1^, Jihong Han^1,4^, Xiaoxiao Yang^1,^*

**Supplementary figures (the whole uncropped images of the original Western blot)**

**Figure 2D**

**
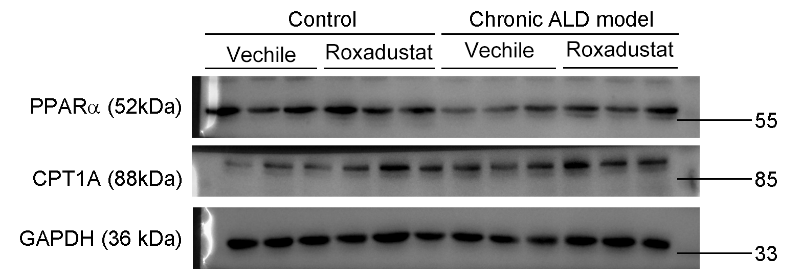
**

**Figure 2E**

**
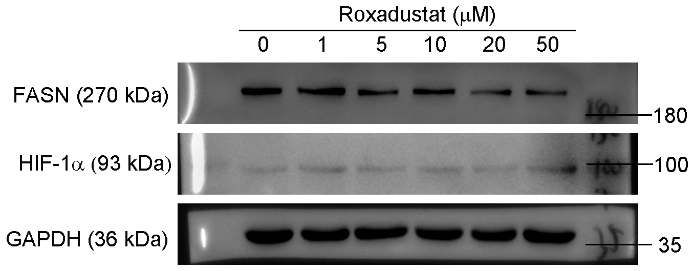
**

**Figure 2F**

**
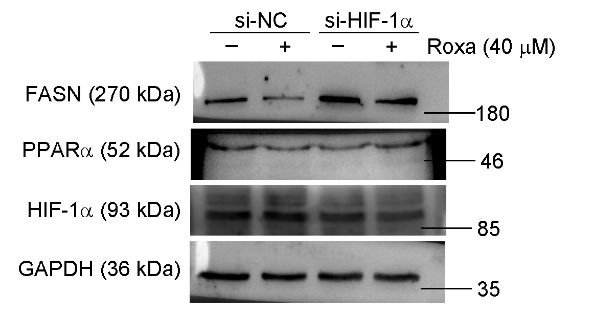
**

**Figure 3A**

**
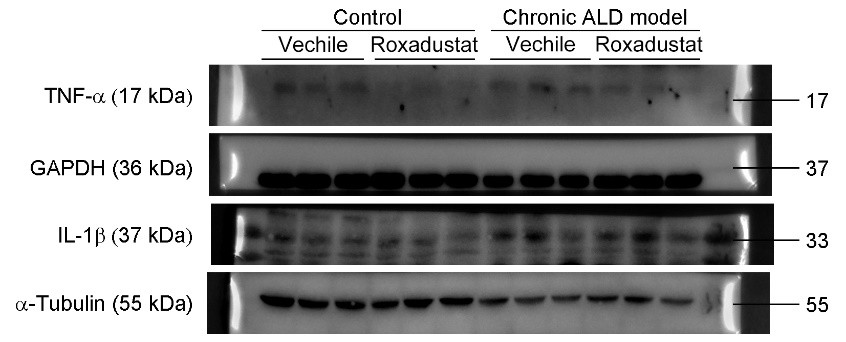
**

**Figure 5D**

**
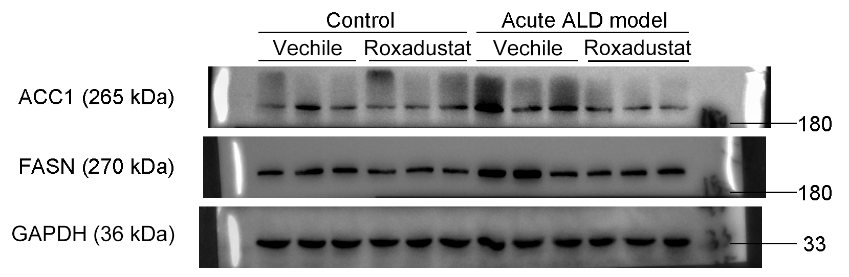
**

**Figure 5E**

**
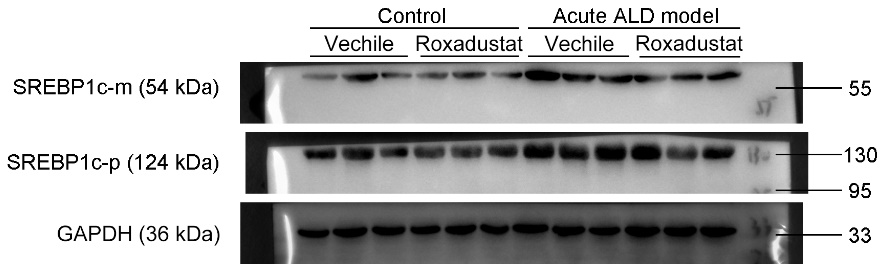
**

**Figure 5F**

**
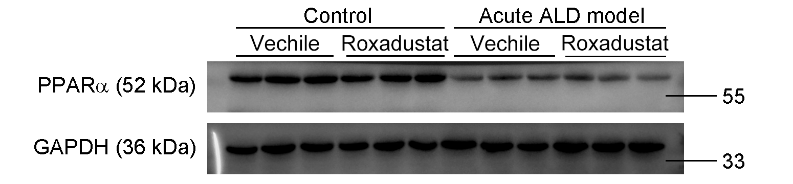
**

**Figure 5G**

**
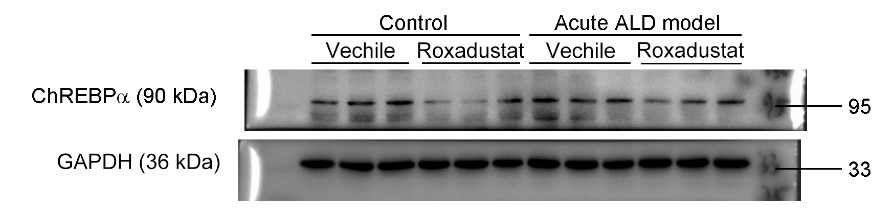
**

**Figure 6B**

**
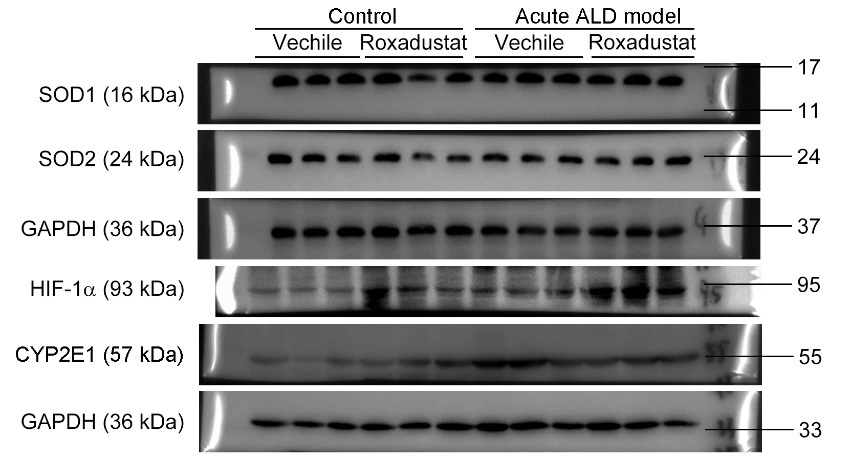
**

**Figure 6C**

**
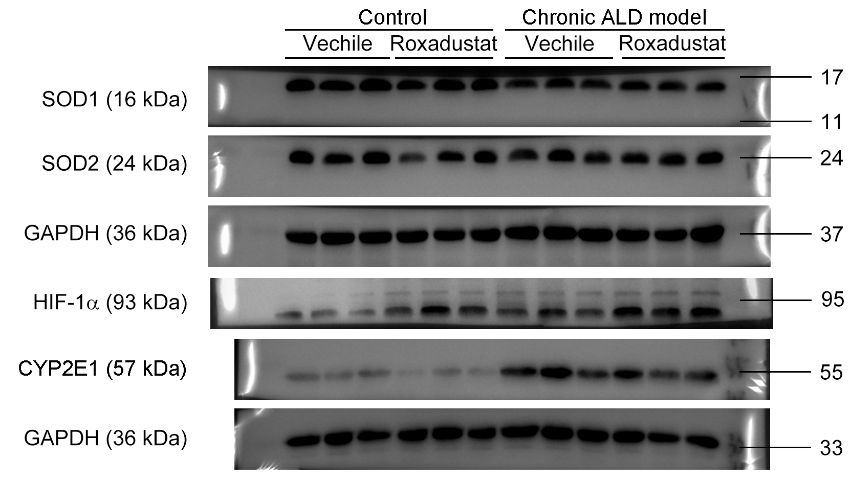
**
