# Supplementary material for: Roxadustat, a Hypoxia-Inducible Factor 1α Activator, Attenuates Both Long- and Short-Term Alcohol-Induced Alcoholic Liver Disease
Source: Front Pharmacol. 2022 May 10;13:895710. doi: 10.3389/fphar.2022.895710 (PMC9127324; doi:10.3389/fphar.2022.895710)
Supplement: Supplementary file 2 [file DataSheet1.docx]

**Supporting Information**

Roxadustat, a hypoxia-inducible factor 1α activator, attenuates both long- and short-term alcohol induced alcoholic liver disease

Yongyao Gao^1,†^, Xiaomeng Jiang^2^, Daigang Yang^1,†^, Wentong Guo^1^, Dandan Wang^3^, Ke Gong^1^, Ying Peng^1^, Hong Jiang^2^, Cunyuan Shi^2^, Yajun Duan^1^, Yuanli Chen^1^, Jihong Han^1,4^, Xiaoxiao Yang^1,^*

**Content**

[Supplementary Figure 1 1](#_Toc91683062)

[Supplemental experimental procedures 2](#_Toc91683063)

[Reference 2](#_Toc91683064)


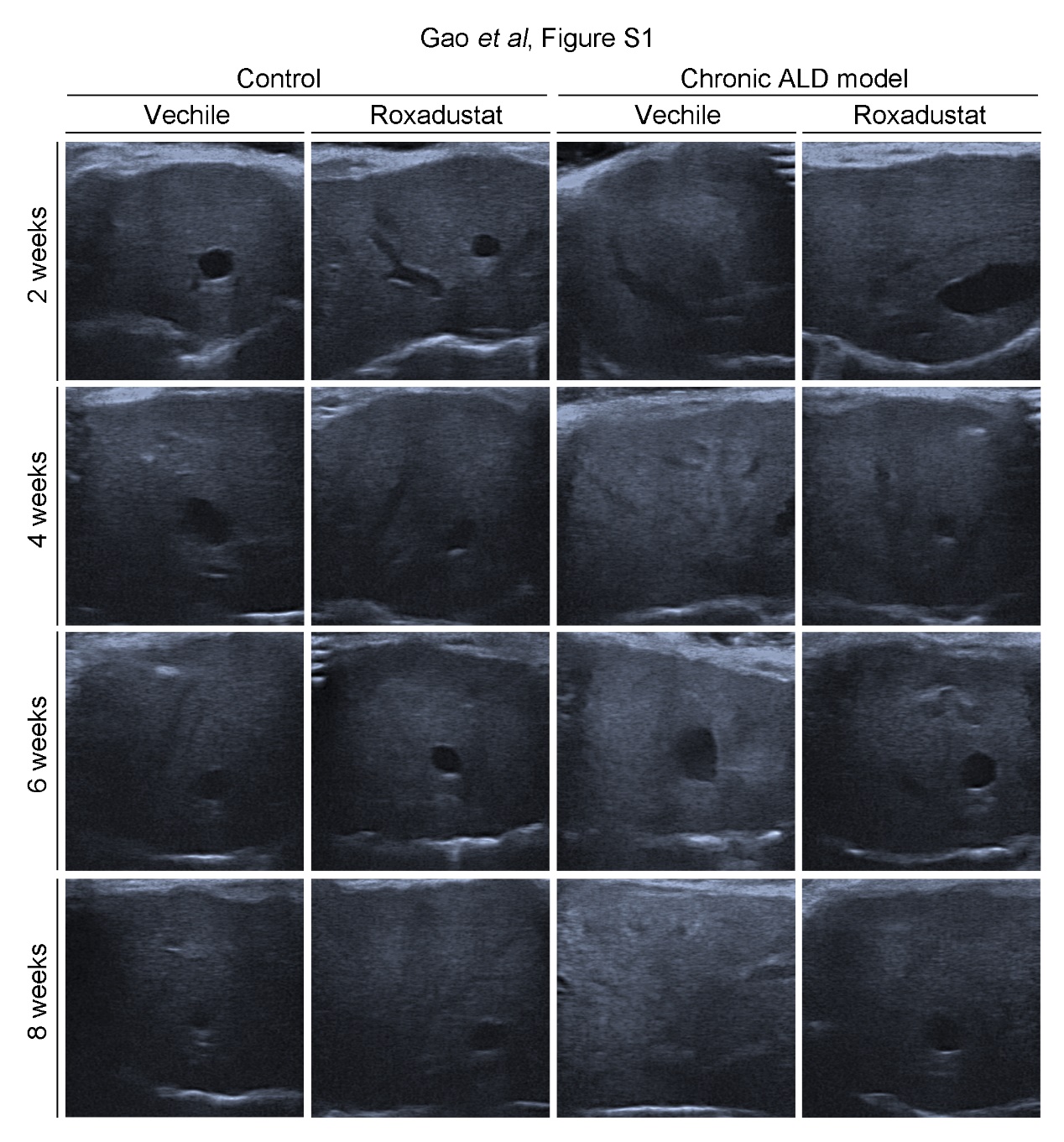


Supplementary Figure 1. **Roxadustat attenuates the development of fatty liver in chronic ALD mouse model.**

Liver ultrasound images of mouse from chronic ALD groups were recorded by an animal specific ultrasound apparatus (VINNO6 System) at 2, 4, 6, 8 weeks.

# Supplemental experimental procedures

**Liver ultrasonography**

Fatty liver was recorded by ultrasonography using an animal specific ultrasound apparatus (VINNO6 System). Mice were anesthetized with 3% isoflurane/oxygen mixture and placed on a heated platform to maintain body temperature. After removing the hair from the abdomen, a probe moistened with coupling agent was detected the abdominal liver of the mice. The liver ultrasound images of each group were recorded at 2, 4, 6, 8 weeks(Mathiesen et al., 2002; Pandit et al., 2019; Zhao et al., 2020).

# Reference

Mathiesen, U., Franzén, L., Aselius, H., Resjö, M., Jacobsson, L., Foberg, U., et al. (2002). Increased liver echogenicity at ultrasound examination reflects degree of steatosis but not of fibrosis in asymptomatic patients with mild/moderate abnormalities of liver transaminases. *Digestive and liver disease : official journal of the Italian Society of Gastroenterology and the Italian Association for the Study of the Liver* 34(7)**,** 516-522. doi: 10.1016/s1590-8658(02)80111-6.

Pandit, H., Tinney, J., Li, Y., Cui, G., Li, S., Keller, B., et al. (2019). Utilizing Contrast-Enhanced Ultrasound Imaging for Evaluating Fatty Liver Disease Progression in Pre-clinical Mouse Models. *Ultrasound in medicine & biology* 45(2)**,** 549-557. doi: 10.1016/j.ultrasmedbio.2018.10.011.

Zhao, D., Xue, C., Li, J., Feng, K., Zeng, P., Chen, Y., et al. (2020). Adiponectin agonist ADP355 ameliorates doxorubicin-induced cardiotoxicity by decreasing cardiomyocyte apoptosis and oxidative stress. *Biochemical and biophysical research communications* 533(3)**,** 304-312. doi: 10.1016/j.bbrc.2020.09.035.
